# Creatine plus β-Hydroxy-β-Methylbutyrate supplementation is associated with preserved glutathione redox-balance and redox–function associations in older adults: a secondary analysis of a randomized crossover trial

**DOI:** 10.1007/s10522-026-10407-2

**Published:** 2026-02-19

**Authors:** Rafael Ramos-Hernández, Juan Mielgo-Ayuso, Diego Fernández-Lázaro, Alba Abia, Juan F. Pérez-López, Miriam Saiz-Rodríguez, Natalia Busto

**Affiliations:** 1 Faculty of Health Sciences, University of Burgos (UBU), 09001 Burgos, Spain; 2 Advanced Research in Integrative Physiology for Life (IAFIV) Research Group, University of Burgos (UBU), 09001 Burgos, Spain; 3 Research Group “Nutrition and Physical Activity”, Spanish Nutrition Society “SEÑ”, 28010 Madrid, Spain; 4 Area of Histology, Faculty of Health Sciences, University of Valladolid, 42004 Soria, Spain; 5 Neurobiology Research Group, Faculty of Medicine, University of Valladolid, 47005 Valladolid, Spain; 6 Faculty of Health Sciences, Universidad Europea Miguel de Cervantes, 47012 Valladolid, Spain

**Keywords:** Oxidative stress, Glutathione, Redox balance, Aging, Creatine, HMB, Secondary analysis

## Abstract

Oxidative stress contributes to age-related musculoskeletal decline, partly through disruption of glutathione-dependent redox homeostasis. Although creatine and β-hydroxy-β-methylbutyrate (HMB) have been individually linked to antioxidant and cytoprotective effects, their combined influence on systemic redox balance in older adults remains insufficiently characterized.To examine the effects of creatine plus HMB supplementation on oxidative stress biomarkers and composite redox indices, and to explore whether redox adaptations co-vary with changes in functional measures in physically active older adults.In a randomized, double-blind, placebo-controlled crossover trial, 30 physically active older adults (62.7 ± 5.3 years; 20 men, 10 women) completed two 6-week intervention phases (3 g/day creatine + 3 g/day calcium HMB vs. placebo) during supervised exercise training. Primary endpoints were oxidized glutathione and the Glutathione Redox Index. Secondary biomarkers and composite indices were analyzed with false discovery rate (FDR) control. Percent changes (Δ%) in functional tests were examined exclusively as exploratory correlates of redox adaptations.Supplementation was associated with attenuation of the placebo-related increase in oxidized glutathione and nominal preservation of the Glutathione Redox Index, although these effects did not remain significant after FDR adjustment. In men, a nominal increase in malondialdehyde was observed under supplementation. Exploratory analyses indicated weak associations between changes in composite redox indices and Δ% functional measures.Creatine plus HMB supplementation was associated with nominal modulation of glutathione-centered redox balance during training in active older adults. Exploratory redox–function associations support further investigation in larger, adequately powered trials.

## Introduction

Aging is characterized by progressive declines in skeletal muscle quality and functional capacity that diminish mobility and quality of life (Cruz-Jentoft et al. 2019). Even in physically active older adults, subtle but relevant decreases in neuromuscular performance may occur despite regular training (Seals et al. 2016), highlighting the need for complementary strategies that support physiological resilience.

Oxidative stress is recognized as a central mechanism contributing to age-related impairments in muscle function. With advancing age, the imbalance between reactive oxygen species (ROS) production and antioxidant defenses leads to cumulative oxidative damage to lipids, proteins, and nucleic acids (Fulle et al. 2004; Ji 2015). Such redox dysregulation has been associated with lower strength, impaired endurance, and reduced adaptive responses to exercise (Powers et al. 2011). Although individual biomarkers (e.g., malondialdehyde, protein carbonyls, glutathione concentrations, antioxidant enzyme activity) offer valuable insights, they represent only fragments of a broader redox network. Accordingly, the use of composite indices integrating oxidative and antioxidant signals is increasingly recommended to characterize systemic redox balance more comprehensively (Jones 2006; Sies 2015; Pisoschi et al. 2021; Alhaj Sulaiman and Katanaev 2025).

Nutritional strategies such as creatine and β-hydroxy-β-methylbutyrate (HMB) have gained attention for their potential to support musculoskeletal health in older adults. Creatine supplementation enhances phosphocreatine availability and training adaptations and may also exert cytoprotective effects through membrane stabilization and attenuation of oxidative insults (Sestili et al. 2006; Riesberg et al. 2016; Candow et al. 2019). HMB, provided in this study as the calcium salt, reduces proteolysis, supports muscle remodeling, and has been linked to modulation of inflammatory and oxidative pathways (Aversa et al. 2011; Fitschen et al. 2013).

A growing body of evidence indicates that HMB may influence oxidative metabolism and mitochondrial function, with meta-analytic data showing improvements in endurance performance and aerobic capacity (Fernández-Landa et al. 2024). Beyond its individual effects, the combination of creatine and HMB has been reported to exert synergic influences on muscle adaptation, hormonal responses, muscle damage markers, and performance across different populations (Fernández-Landa et al. 2019, 2020a, b). Importantly, functional strength improvements following creatine plus HMB supplementation were recently documented in this same cohort during combined exercise training (Ramos-Hernández et al. 2025) raising the question of whether redox modulation may contribute to these adaptations.

Despite these advances, to our knowledge, no study has comprehensively examined whether creatine plus HMB modulates systemic oxidative stress or composite glutathione-based redox indices in physically active older adults, nor whether redox shifts relate to functional adaptations previously observed. This knowledge gap is relevant because redox regulation may influence both muscle contractile function and adaptation to exercise.

To address this gap, we conducted a randomized, double-blind, placebo-controlled crossover trial to evaluate the effects of six weeks of creatine plus calcium HMB supplementation on oxidative stress biomarkers and composite redox indices. The primary objective was to assess supplementation effects on glutathione status—oxidized glutathione (GSSG) and the Glutathione Redox Index (GRI). A secondary exploratory objective was to assess whether changes in redox indices were associated with changes in muscle strength and functional performance, whose absolute values were previously published for this cohort. Given the high inter-individual variability characteristic of redox biology, a crossover design was chosen to enhance statistical sensitivity (Jones and Kenward 2014).

We hypothesized that creatine plus calcium HMB would (i) preserve glutathione redox balance by attenuating increases in GSSG and improving composite indices, and (ii) exhibit modest associations between redox shifts and functional changes.

## Materials and methods

### Study design and participants

This study reports a prespecified secondary analysis of a randomized, double-blind, placebo-controlled crossover trial involving 30 physically active older adults (62.7 ± 5.3 years; range 60–82; 20 men and 10 women) recruited from senior centers and community sports programs in Tenerife, Spain. The parent trial evaluated the effects of creatine monohydrate plus β-hydroxy-β-methylbutyrate in the calcium salt form (HMB–Ca) combined with supervised exercise training. The present analysis focuses specifically on oxidative stress biomarkers, composite redox indices, and their exploratory associations with previously published functional outcomes.

#### Sample size considerations

Formal a priori power calculations for crossover trials require reliable estimates of within-subject variances for the specific primary endpoints—in this case, GSSG and the Glutathione Redox Index (GRI). Because no validated variance data were available for these biomarkers in physically active older adults, a formal a priori power calculation could not be performed without relying on speculative assumptions.

Instead, the recruitment target was based on established methodological benchmarks for two-period crossover trials, which consistently show that 24–30 participants provide adequate power to detect small-to-moderate within-subject effects, while taking advantage of the efficiency gains inherent to this design (Senn 2003; Jones and Kenward 2014). Following this guidance, 30 participants were recruited to ensure robust estimation of redox endpoints and to accommodate potential attrition. All 30 participants completed both intervention phases.

To complement this justification, a post-hoc sensitivity analysis was conducted. Based on the observed within-subject effect sizes for the primary endpoints (η^2^p = 0.17–0.42), the achieved sample yielded ≥ 78% statistical power, confirming that the realized sample size was adequate for detecting the effects actually observed in this cohort.

This hybrid approach—benchmark-based a priori justification plus post-hoc sensitivity assessment—is consistent with accepted practices for crossover trials in emerging biomarker fields, where reliable variance estimates are scarce.

#### Randomization and blinding

Participants were stratified by sex and randomized (1:1) into two treatment sequences using a computer-generated allocation prepared by an independent researcher. Supplements and placebo were packaged in identical coded sachets to ensure allocation concealment. Participants, investigators, and outcome assessors remained blinded until completion of data analysis.

#### Timeline and assessments

**Fig. 1:**
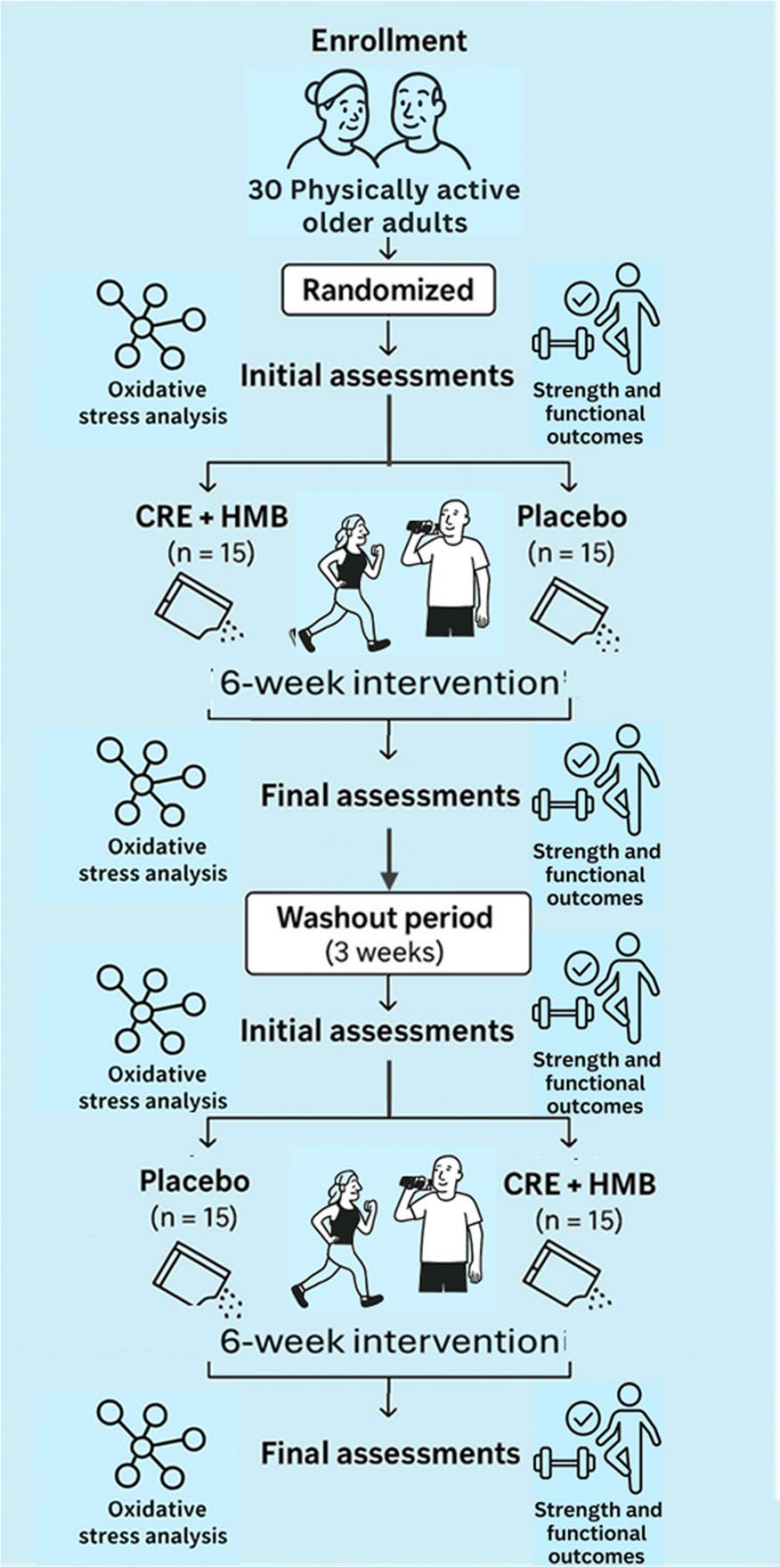
Study flowchart of the randomized, double − blind, placebo − controlled crossover trial CRE + HMB, creatine plus β − hydroxy − β − methylbutyrate

The intervention consisted of two 6-week supplementation phases separated by a 3-week washout. Assessments were conducted at four time points: T1 (baseline, pre-phase 1), T2 (post-phase 1), T3 (pre-phase 2, post-washout), and T4 (post-phase 2). For the primary crossover comparisons, T1 and T3 were pooled as PRE, and T2 and T4 as POST, following standard crossover analytical procedures to enhance robustness against biological variability. The 3-week washout was considered sufficient based on prior moderate-dose creatine trials without loading (Fig. 1).

#### Eligibility criteria

Inclusion criteria were: age ≥ 60 years; engagement in ≥ 150 min/week of structured physical activity for ≥ 6 months; and absence of severe cardiometabolic, renal, hepatic, or musculoskeletal disorders. Exclusion criteria included recent use of creatine, HMB, or other ergogenic supplements; advanced chronic disease; or recent osteoporotic fracture. All participants were community-dwelling, physically active older adults.

#### Ethics and registration

The study was approved by the University of Burgos Ethics Committee (IR 24/2023) and registered at ClinicalTrials.gov (NCT05951439). All participants provided written informed consent.

### Supplementation protocol

During each intervention phase, participants received either 3 g/day of creatine monohydrate plus 3 g/day of β-hydroxy-β-methylbutyrate in the calcium salt form (HMB-Ca) or a matched placebo (6 g/day of inulin). All raw materials—creatine monohydrate, HMB-Ca, and inulin—were sourced from the same commercial supplier (HSN, Spain). The research team weighed and pre-packaged the ingredients into identical opaque 6-g sachets, ensuring indistinguishable appearance, texture, and flavor across conditions. Inulin was selected as placebo due to its inert metabolic profile and lack of known effects on muscle or inflammatory markers.

Participants consumed one sachet daily, dissolved in yogurt or fruit juice, preferably in the evening to standardize timing and minimize potential interference with meals or training sessions. A licensed dietitian-nutritionist provided written and verbal instructions to ensure consistency across participants. Supplement boxes containing 42 sachets (corresponding to 6 weeks) were distributed at T1 and T3.

Adherence was monitored weekly through self-reported logs, sachet counts, and direct supervision by research staff. Compliance exceeded 95%, and no gastrointestinal, musculoskeletal, or other adverse events were reported, consistent with previous evidence supporting the safety of creatine and HMB-Ca in older adults (Ramos-Hernández et al. 2025).

The dosage and timing were selected based on previous trials demonstrating efficacy and safety of creatine and HMB-Ca in both athletic populations (Fernández-Landa et al. 2019, 2020a, b), and older adults (Ramos-Hernández et al. 2025).

### Nutritional evaluation

To minimize potential dietary confounding, all participants received individualized guidance from a licensed dietitian-nutritionist aimed at maintaining stable energy and protein intake throughout the intervention. Recommendations were aligned with current guidelines for active older adults, encouraging consumption of ≥ 1.2 g/kg/day of protein and approximately 35–40 kcal/kg/day of total energy intake (Bauer et al. 2013).

Dietary habits were monitored at the end of each 6-week phase using a validated Food Frequency Questionnaire (FFQ) (Martin-moreno et al. 1993) which assessed habitual intake over the preceding month across 24 food categories (including dairy products, meat, fish, cereals, fruits, vegetables, fats, beverages, and processed foods). The FFQ was used to verify dietary consistency across conditions, rather than to quantify absolute intake. No meaningful differences in estimated energy intake, macronutrient distribution, or predominant food group consumption were observed between the CRE + HMB and placebo phases, indicating that dietary patterns remained stable throughout the study.

### Integral physical conditioning (IPC) program

All participants followed the same individualized IPC program during both intervention phases. Training consisted of four supervised sessions per week (60 min each) with overall adherence ≥ 90%. Each session followed a three-part structure (warm-up, main part, cooldown) aligned with ACSM recommendations for older adults, with the primary goal of enhancing functional fitness and preserving independence through a multicomponent approach (strength, power, cardiovascular endurance, balance, coordination, mobility, flexibility) (Chodzko-Zajko et al. 1998).

Periodization. The IPC was organized into two 6-week blocks separated by the 3-week washout. Each 6-week block comprised two mesocycles of three microcycles (weeks). The first microcycle emphasized familiarization (higher volume, lower intensity), followed by progressive increases in load and task complexity in subsequent microcycles. This model ensured early neuromuscular adaptation and safe workload progression while maximizing functional transfer in later phases, consistent with our previous trial in the same population (Ramos-Hernández et al. 2025).

Intensity prescription and monitoring. Exercise intensity was individualized using objective and subjective markers. Training heart rate (THR) was calculated via the Karvonen formula; strength loads were prescribed from estimated 1RM using the Brzycki equation. Both were mapped to a modified Borg scale to harmonize internal load across modalities (e.g., THR/1RM ≈ 100% → RPE 10; 90% → 9; 80% → 8; … 40% → 4). Session training load was monitored through session-RPE, number of exercises, repetitions, and total duration; weekly training logs were verified by supervisors. Adherence was ≥ 90% across participants.

Session components.

- Warm-up (5–12 min): dynamic mobility, coordination, balance and low-intensity drills to activate cardiometabolic and neuromuscular systems.
- Main part (20–50 min): integrated modalities delivered as single-focus sessions or combined within multicomponent formats:
  o Strength training: multi- and single-joint exercises for all major muscle groups using free weights, machines, bands or bodyweight. Loads typically progressed from 50–60% 1RM (introductory weeks) towards 60–90% 1RM in load microcycles; 2–4 sets × 6–12 reps, with 1–3 min rests, prioritizing large over small muscle groups and alternating global/auxiliary movements.
  o Power training: high-velocity movements (medicine-ball throws, jumps, ballistic lifts). Typical intensities 20–50% 1RM initially, progressing to ≥ 60–80% 1RM in advanced phases; 2–5 sets × 1–5 reps, rests 30 s–3 min, individualized to peak power output.
  o Multicomponent circuits (MCC): 6–12 stations mixing global strength, aerobic endurance, balance, coordination, agility and mobility; progression via load increments, reduced recovery, and technical complexity. Nine standardized MCC sessions were rotated across microcycles (see Supplementary Table S1 for full sequences and progression).
  o High-Intensity Interval Training (HIIT): bouts at 80–100% THR or ≥ 80% 1RM (for strength-power tasks) with 20–90 s recoveries; 4–8 work intervals per block, 2–4 blocks per session, 2–3 min recovery between blocks. Four HIIT formats were used uniformly: HIIT-Core, HIIT-Global, HIIT-Full Body, HIIT-Power, targeting complementary capacities.
  o Moderate-Intensity Continuous Training (MICT): continuous or interval aerobic work at 40–60% THR (e.g., brisk walking, cycling, rowing, elliptical), 1–4 sets × 3–20 min, with minimal inter-set recovery; progression via longer bouts, interval inclusion, or modality combinations while maintaining moderate intensity.
- Cooldown (5–10 min): low-intensity movements, static/dynamic stretching; when appropriate, PNF, foam-rolling and guided breathing for recovery.

Standardization and safety. Exercise delivery and progression were standardized across participants, supervised by qualified staff, and adapted to individual capabilities to minimize injury risk. No training-related adverse events were recorded. The IPC structure, intensity progressions, and multicomponent emphasis replicate the program previously validated in this cohort (Ramos-Hernández et al. 2025) and conform to guideline-based prescriptions for older adults.

### Outcome measures

#### Oxidative stress biomarkers

##### Blood collection and pre-analytical control

Venous blood was obtained at each assessment point (T1–T4) after an overnight fast (≥ 10 h), between 08:00 and 10:00 h, with participants seated for ≥ 10 min. To minimize acute redox fluctuations, participants were instructed to avoid vigorous exercise for 48 h and alcohol for 24 h, while maintaining their usual medications. Samples were collected into sodium citrate tubes (plasma) and serum separator tubes (serum), centrifuged within 60 min (1500–2000 g, 10–15 min, 4 °C), aliquoted (200–500 µL), and stored at − 80 °C until analysis (single freeze–thaw). Hemolysis was evaluated visually and by hemolysis index; samples exceeding pre-specified thresholds were excluded. For each participant, PRE and POST samples were analyzed in the same run/plate. Laboratory staff were blinded to allocation and timepoint.

##### Protein quantification and normalization

Total protein concentration was determined by the Bradford method, using bovine serum albumin (BSA, mg/mL) as standard. Protein content was used to normalize assays when applicable (e.g., FRAP, µM/mg protein; fluorescent oxidation products, a.u./µg plasma). In the dataset, “BSA (mg/mL)” represents the albumin-equivalent calibration used in colorimetric assays and was retained as the reference measure of total protein concentration for normalization and quality control.

##### Analytical methods and quality control

All biomarkers were measured in duplicate using validated spectrophotometric or fluorometric assays previously established in our laboratory (Barrenetxea-Garcia et al. 2025). Samples with intra-assay coefficients of variation > 10% were re-analyzed. Each plate included blanks, calibration standards, and an internal pooled control. Unless otherwise indicated, results are reported as plasma concentrations or normalized to protein content when applicable.

For interpretative clarity, biomarkers were conceptually grouped into domains reflecting protein normalization, antioxidant capacity, glutathione redox system, and oxidative damage, consistent with the composite redox indices defined below:

- Ferric reducing antioxidant power (FRAP, µM/mg): reduction of Fe^3^⁺–TPTZ to Fe^2^⁺–TPTZ at 593 nm using a microplate reader.
- Protein carbonyls (mM): DNPH derivatization with spectrophotometric detection at 370–375 nm.
- Reduced glutathione (GSH, µM) and oxidized glutathione (GSSG, µM): Both biomarkers were quantified using a fluorometric OPA assay, with N-ethylmaleimide (NEM) pre-treatment for selective GSSG measurement. Fluorescence was recorded at λ_exc = 360 nm and λ_em = 460 nm. Concentrations were calculated from standard curves prepared with purified GSH and GSSG and are reported in micromolar units (µM). All samples were analyzed in the same batch to ensure comparability. The GSH/GSSG ratio was subsequently computed.
- Total thiols (µM): Ellman’s reagent (DTNB) at 412 nm using a microplate reader.
- Reactive oxygen and nitrogen species (ROS/RNS, RFU/µg protein): quantified using 2’,7’-dichlorodihydrofluorescein diacetate (DCFH₂-DA) as a fluorescent probe, with excitation/emission at 485/520 nm, normalized by protein load. This assay reflects overall oxidative activity in plasma rather than specific ROS species. (RFU = relative fluorescence units).
- Glutathione peroxidase (GPx, U/mL): coupled assay monitoring NADPH oxidation at 340 nm using a microplate reader.
- Malondialdehyde (MDA, nM): TBARS assay with thiobarbituric acid, with absorbance read at 530 nm using a microplate reader. Results are expressed as nM equivalents of 1,1,3,3-tetramethoxypropane.

##### Rationale

This panel integrates enzymatic and non-enzymatic antioxidants with oxidative damage markers, allowing a comprehensive evaluation of systemic redox balance in older adults under exercise and supplementation interventions.

##### Data handling

Variables with skewed distributions (e.g., MDA, GSSG, RFU) were log-transformed before statistical analysis, although descriptive statistics are presented in the original scale.

##### Composite redox indices

To integrate the biomarker panel, standardized z-scores were calculated using the PRE distribution of the total sample as reference (mean ± SD), ensuring that standardization was independent of POST values. For sensitivity analyses, sex-specific PRE distributions were also tested, yielding comparable results. Composite indices were then derived as follows:

- Antioxidant capacity index (ACI) = z(FRAP) + z(GPx) + z(Total thiols) + z(GSH).
- Oxidative damage index (ODI) = z(MDA) + z(Protein carbonyls) + z(Fluorescent oxidation products).
- Glutathione redox index (GRI) = calculated as the natural logarithm of the GSH/GSSG ratio [ln(GSH/GSSG)].
- Redox balance index (RBI) = ACI – ODI

Standardization was performed according to: z_i_ = (X_i_—μ_PRE_) / σ_PRE_.

where X_i_ is the individual biomarker value, and μ_PRE_, σ_PRE_ are the mean and standard deviation of the PRE distribution for the total sample.

These indices have been proposed in the literature as integrative tools to capture systemic oxidative balance (Jones 2006; Pisoschi et al. 2021; Alhaj Sulaiman and Katanaev 2025). This strategy provides a more comprehensive overview of redox status than isolated biomarkers, which are often subject to variability and context-dependent interpretation.

#### Muscle strength and functional performance

A battery of standardized physical function tests was administered as part of the parent trial, following international recommendations (Thompson et al. 2013; Dent et al. 2019), and detailed methodology has been published previously (Ramos-Hernández et al. 2025). In the present study, these measures were not analyzed as efficacy outcomes. Instead, they were used exclusively to derive percent changes (Δ%) for exploratory associations with redox biomarkers.

Upper- and lower-limb strength were assessed in the parent trial using handgrip dynamometry, leg/back dynamometry, and the 30-s arm-curl test. Muscular endurance was evaluated using the 30-s push-up and 30-s crunch tests. Functional performance was assessed using 4-m gait speed, the 5-repetition chair-stand test, Timed Up and Go (TUG), the 400-m walk test, and standardized static balance positions. Global physical performance was summarized using the Short Physical Performance Battery (SPPB).

All assessments were performed under standardized conditions at baseline and post-intervention, with PRE defined as pooled T1–T3 and POST as pooled T2–T4, consistent with the crossover design. For the present analyses, only percent changes (Δ%) were used, and no absolute values or efficacy interpretations are reported, as these have been previously published for this cohort (Ramos-Hernández et al. 2025). Sex-stratified Δ% values were used exclusively for exploratory associations with redox variables.

### Statistical Analysis

#### Endpoints

The primary endpoints were oxidized glutathione (GSSG) and the Glutathione Redox Index (GRI), which were prespecified due to their sensitivity to redox shifts. Secondary endpoints included all remaining oxidative stress biomarkers and composite indices (ACI, ODI, RBI). Measures of muscle strength and functional performance were not analyzed as outcomes of this study; instead, only percent changes (Δ%) from these tests were used as exploratory variables to examine associations with redox adaptations.

#### Data structure and crossover validation

Data from the four assessments (T1–T4) were collapsed into PRE (T1 + T3) and POST (T2 + T4) to minimize intra-individual variability, following established methodological guidance for two-period crossover trials (Senn 2003; Wellek and Blettner 2012). Carryover and period effects were evaluated through mixed-effects models including Condition (CRE + HMB vs placebo), Time (PRE vs POST), Period, and Sequence as fixed factors, and Participant as a random effect. Sequence × Period interactions and comparisons of T1 vs T3 indicated no evidence of carryover, supporting the pooling strategy.

#### Primary analysis

Supplementation effects on primary and secondary redox endpoints were evaluated using two-way repeated-measures ANCOVA (Time × Condition), with age and PRE values as covariates. Partial eta squared (η^2^p) is reported as an effect size. When assumptions were not fully met, analyses were repeated using percent change (Δ%) values with ANCOVA using the same covariates. Because only two conditions were compared, no post hoc tests were required.

#### Exploratory associations

Associations between Δ redox indices and Δ% functional variables were examined using multivariable linear regression models adjusted for age. Sex-stratified exploratory models were also performed. Diagnostic checks confirmed linearity, homoscedasticity, and normality of residuals. Results are reported as standardized β coefficients with 95% confidence intervals and adjusted R^2^. These analyses are exploratory and are interpreted accordingly.

#### Data handling and multiplicity

Normality was assessed using Shapiro–Wilk tests and Q–Q plots. Skewed variables (e.g., MDA, GSSG, RFU) were log-transformed for inferential analyses; descriptive statistics are presented in the original scale, with back-transformed values reported where appropriate.

Primary endpoints (GSSG, GRI) were evaluated at α = 0.05.

Secondary endpoints were controlled using the Benjamini–Hochberg false discovery rate (FDR) procedure.

Exploratory analyses were not adjusted for multiplicity and should be interpreted cautiously.

All analyses were performed in R v4.3.2 and SPSS v29.

## Results

### Participant flow and baseline characteristics

Formal tests for sequence, period, and carryover effects did not reveal relevant interactions (all p > 0.10), supporting the validity of the crossover design assumptions. A total of 40 older adults were initially recruited, of whom 30 (20 men and 10 women; mean age 62.7 ± 5.3 years, range 60–82) completed all phases of the crossover trial. Overall adherence to the supplementation protocol and to the supervised integral physical conditioning (IPC) program exceeded 90%. No supplementation- or training-related adverse events were reported; only mild, transient muscle soreness was noted during the early training sessions.

At baseline, no meaningful differences were observed between the two randomization sequences (CRE + HMB first vs. placebo first) in age, sex distribution, anthropometry, oxidative stress biomarkers, or physical function tests (data not shown), confirming the adequacy of random allocation.

### Oxidative stress biomarkers

To provide a comprehensive evaluation of systemic redox status, individual oxidative stress biomarkers are presented in Table 1, organized by physiological domains, while composite redox indices integrating these biomarkers are summarized in Table 2.

**Table 1:** Oxidative stress biomarkers at PRE (before each intervention period) and POST (after supplementation with creatine plus HMB or placebo) in physically active older adults

Biomarker	Time	Total sample (*n* = 30)				Male (*n* = 20)			Female (*n* = 10)				
		CRE + HMB	PLA	P (η^2^p)	FDR	CRE + HMB	PLA	P (η^2^p)	FDR	CRE + HMB	PLA	P (η^2^p)	FDR
Control / normalization													
Total protein (mg/mL)	PRE	72.98 ± 20.44	81.55 ± 22.92	0.212 (0.155)	0.732	72.17 ± 21.29	83.66 ± 22.91	0.178 (0.233)	0.445	74.60 ± 19.63	77.33 ± 23.55	0.75 (0.037)	0.947
	POST	56.03 ± 8.65	57.91 ± 7.36			56.21 ± 10.01	59.14 ± 5.66			55.65 ± 5.41	55.44 ± 9.85		
Antioxidant capacity (→ ACI)													
FRAP (µM/mg)	PRE	6.78 ± 5.74	5.88 ± 2.38	0.615 (0.059)	0.732	6.33 ± 1.55	6.30 ± 2.26	0.427 (0.059)	0.605	7.70 ± 9.97	5.03 ± 2.50	0.338 (0.808)	0.947
	POST	5.11 ± 1.15	4.71 ± 1.06			5.38 ± 1.25	4.84 ± 1.05$			4.55 ± 0.67	4.47 ± 1.10		
GPx (U/mL)	PRE	2100.5 ± 243.1	1997.0 ± 224.1	0.521 (0.012)	0.732	2096.6 ± 241.6	1991.2 ± 212.4	0.375 (0.033)	0.605	2108.1 ± 259.0	2008.4 ± 257.5	0.947 (< 0.001)	0.947
	POST	2156.5 ± 306.7$	2112.6 ± 278.4$			2132.3 ± 301.4$	2122.2 ± 251.2$			2204.9 ± 327.5	2093.4 ± 340.6$		
Total Thiols (µM)	PRE	39.41 ± 31.59	44.57 ± 31.09	0.987 (< 0.001)	0.987	38.84 ± 22.40	45.34 ± 35.39	0.939 (0.001)	0.939	40.55 ± 46.42	43.03 ± 21.61	0.882 (0.010)	0.947
	POST	40.17 ± 26.83	45.48 ± 24.45			38.82 ± 24.04	44.48 ± 26.31			42.87 ± 32.97	47.50 ± 21.43		
GSH (µM)	PRE	243.54 ± 83.03	224.86 ± 72.58	0.542 (0.017)	0.732	231.40 ± 78.65	215.89 ± 70.60	0.675 (0.012)	0.750	267.81 ± 90.41	242.78 ± 76.90	0.628 (0.036)	0.947
	POST	220.62 ± 82.60$	185.98 ± 65.67			220.13 ± 87.43	190.99 ± 70.26			221.61 ± 76.45$	175.95 ± 57.53		
Glutathione redox system (→ GRI)													
GSSG (µM)	PRE	44.02 ± 14.32	43.50 ± 13.06	0.039 (0.097)	0.390	44.66 ± 14.99	41.12 ± 12.95	0.049 (0.129)	0.245	42.72 ± 13.57	48.25 ± 12.58	0.582 (0.028)	0.947
	POST	39.66 ± 10.45*$	48.07 ± 18.26			39.49 ± 10.59*$	48.04 ± 21.23			40.01 ± 10.72	48.14 ± 11.08		
GSH/GSSG ratio	PRE	6.72 ± 4.06	5.79 ± 2.83	0.506 (0.024)	0.732	6.39 ± 4.13	5.92 ± 3.00	0.484 (0.036)	0.605	7.37 ± 4.06	5.53 ± 2.59	0.894 (0.005)	0.947
	POST	6.31 ± 3.59	4.61 ± 2.58$			6.38 ± 3.88	4.87 ± 2.70$			6.17 ± 3.12	4.08 ± 2.35		
Oxidative damage (→ ODI)													
Protein Carbonyls (mM)	PRE	0.61 ± 0.09	0.57 ± 0.11	0.291 (0.034)	0.732	0.62 ± 0.10	0.59 ± 0.10	0.231 (0.066)	0.462	0.58 ± 0.07	0.53 ± 0.13	0.770 (0.011)	0.947
	POST	0.60 ± 0.11	0.60 ± 0.10$			0.58 ± 0.10	0.60 ± 0.09$			0.63 ± 0.12	0.60 ± 0.14		
FOPs (a.u./µg plasma)	PRE	84.50 ± 52.07	89.68 ± 88.15	0.659 (0.034)	0.732	85.36 ± 29.40	108.74 ± 102.99	0.152 (0.383)	0.445	82.78 ± 83.10	51.55 ± 15.04	0.140 (0.010)	0.947
	POST	60.83 ± 22.17	59.16 ± 21.27			68.82 ± 22.89	64.86 ± 21.72			44.87 ± 7.28$	47.75 ± 15.65		
MDA (nM)	PRE	1.76 ± 1.37	1.65 ± 1.39	0.392 (0.015)	0.732	1.56 ± 0.98	1.52 ± 1.40	0.035 (0.118)	0.245	2.20 ± 1.98	1.92 ± 1.38	0.385 (0.030)	0.947
	POST	3.49 ± 2.10	2.92 ± 2.47			3.73 ± 2.07*	2.49 ± 1.78			3.01 ± 2.19	3.79 ± 3.42		

Data are expressed as mean ± SD

*FRAP* ferric reducing antioxidant power, *GSH* reduced glutathione, *GSSG* oxidized glutathione, *GPx* glutathione peroxidase, *MDA* malondialdehyde, *FOPs* fluorescent oxidation products (a.u./µg plasma)

P values correspond to the time × condition interaction in mixed − effects models adjusted for age and PRE values. When models did not converge, Δ values (POST − PRE) were analyzed using ANCOVA adjusted for age and PRE. Η^2^p indicates partial eta squared

^*^ Indicates a significant between − condition difference at POST (*p* < 0.05). ^$^ Indicates a significant within − condition PRE–POST change (*p* < 0.05)

FDR − adjusted p values were calculated using the Benjamini–Hochberg procedure

**Table 2:** Oxidative stress indices at PRE (before each intervention period) and POST (after supplementation with creatine plus HMB or placebo) in physically active older adults

Index	Time	Total Sample (*n* = 30)				Male (*n* = 20)				Female (*n* = 10)			
		CRE + HMB	PLA	P (η^2^p)	FDR	CRE + HMB	PLA	P Η^2^p	FDR	CRE + HMB	PLA	P Η^2^p	FDR
ACI	PRE	0.60 ± 2.55	− 0.12 ± 1.69	0.766 (0.005)	0.986	0.27 ± 1.61	− 0.10 ± 1.50	0.989 (< 0.001)	0.986	1.26 ± 3.85	− 0.16 ± 2.12	0.664 (0.042)	0.664
	POST	0.03 ± 1.46	− 0.51 ± 1.46			− 0.03 ± 1.47	− 0.41 ± 1.40			0.15 ± 1.51	− 0.72 ± 1.63		
ODI	PRE	0.01 ± 1.49	− 0.33 ± 2.56	0.986 (< 0.001)	0.986	0.04 ± 1.00	0.13 ± 2.92	0.404 (0.054)	0.539	− 0.05 ± 2.33	− 1.24 ± 1.31	0.210 (0.138)	0.664
	POST	0.31 ± 1.63	0.00 ± 1.61			0.42 ± 1.59	− 0.10 ± 1.38			0.09 ± 1.76	0.22 ± 2.05		
GRI	PRE	1.70 ± 0.68	1.63 ± 0.52)	0.213 (0.077)	0.852	1.64 ± 0.68	1.65 ± 0.53	0.269 (0.082)	0.539	1.81 ± 0.72	1.60 ± 0.53	0.555 (0.075)	0.664
	POST	1.68 ± 0.58*	1.34 ± 0.66$			1.68 ± 0.61	1.38 ± 0.73$			1.69 ± 0.56$	1.27 ± 0.53		
RBI	PRE	0.56 ± 2.02	0.21 ± 2.08	0.782 (0.002)	0.986	0.23 ± 1.55	− 0.23 ± 2.11	0.391 (0.032)	0.539	1.30 ± 2.78	1.08 ± 1.80	0.603 (0.032)	0.664
	POST	− 0.28 ± 1.85	− 0.52 ± 1.97			− 0.45 ± 1.88	− 0.30 ± 1.62			0.06 ± 1.82	− 0.94 ± 2.58		

Data are expressed as mean ± SD

P values correspond to the time × condition interaction in mixed − effects models adjusted for age and PRE values. When models did not converge, Δ values (POST − PRE) were analyzed using ANCOVA adjusted for age and PRE. Η^2^p indicates partial eta squared

^*^ Indicates a significant between − condition difference at POST (*p* < 0.05)

^$^ Indicates a significant within − condition PRE–POST change (*p* < 0.05)

FDR − adjusted p values were calculated using the Benjamini–Hochberg procedure

ACI, Antioxidant Capacity Index; GRI, Glutathione Redox Index; ODI, Oxidative Damage Index; RBI, Redox Balance Index

#### Individual oxidative stress biomarkers (Table 1)

Control / normalization: Total protein concentration, used as a normalization and quality-control marker, showed a comparable PRE–POST reduction in both conditions across the total sample and sex-stratified analyses, with no significant Time × Condition interactions (Table 1). This indicates that subsequent biomarker results were not driven by differences in plasma protein content.

Antioxidant capacity (→ ACI): Markers of antioxidant capacity, including FRAP, GPx activity, total thiols, and reduced glutathione (GSH), remained largely stable across conditions in the total sample and by sex (Table 1). No significant Time × Condition interactions were observed for these parameters. Although isolated within-condition PRE–POST changes were detected for GPx activity and total thiols, these changes were comparable between CRE + HMB and placebo and did not survive correction for multiple comparisons.

Glutathione redox system (→ GRI): Within the glutathione redox system, a Time × Condition interaction was observed for oxidized glutathione (GSSG) in the total sample (p = 0.039, η^2^p = 0.097) and in men (p = 0.049, η^2^p = 0.129), although these effects did not remain significant after FDR adjustment (Table 1). Nominal analyses indicated that GSSG increased from PRE to POST under placebo, while remaining relatively stable under CRE + HMB, resulting in lower POST values in the supplemented condition.

The GSH/GSSG ratio showed a parallel, though non-significant, tendency toward more favorable values under supplementation, driven mainly by a decline in the placebo condition. These findings suggest that glutathione-related biomarkers were among the most responsive endpoints to the intervention.

Oxidative damage (→ ODI): Markers of oxidative damage, including protein carbonyls and fluorescent oxidation products (FOPs), remained stable across conditions (Table 1). In contrast, malondialdehyde (MDA) showed a Time × Condition interaction in men (*p* = 0.035, η^2^*p* = 0.118), with higher POST values under CRE + HMB compared with placebo. As this effect did not survive FDR correction and was not observed in women or in the total sample, it should be interpreted as exploratory.

### Composite redox indices

When individual biomarkers were integrated into composite indices, no significant Time × Condition interactions were observed for the Antioxidant capacity index (ACI), Oxidative damage index (ODI), or Redox balance index (RBI) in the total sample or after sex stratification (Table 2).

In contrast, the Glutathione Redox Index (GRI) showed nominally higher POST values under CRE + HMB compared with placebo (raw p < 0.05), reflecting a tendency toward a more reduced glutathione environment with supplementation. Intragroup analyses indicated that this pattern was primarily driven by a decline in GRI under placebo, whereas values were preserved under CRE + HMB. However, these effects did not remain significant after FDR correction and should be considered exploratory.

Taken together, analyses of individual biomarkers and composite indices consistently indicate that creatine plus HMB supplementation did not induce broad changes in systemic antioxidant capacity or oxidative damage. Instead, the most responsive signals were confined to the glutathione redox system, supporting its higher sensitivity as a redox-regulatory domain in the context of nutritional supplementation.

### Associations between redox indices and functional measures

As previously reported for this cohort, CRE + HMB supplementation combined with training improved several strength and functional outcomes (Ramos-Hernández et al. 2025). In the present analysis, we did not re-evaluate efficacy or re-plot these outcomes; instead, percent changes (Δ%) from these tests were used only as exploratory correlates of redox adaptations.

Regression analyses adjusted for age and supplementation group revealed limited but suggestive associations between changes in redox indices and changes in functional measures (Table 3). In the total sample, ΔODI was positively associated with changes in SPPB score (β = 0.299, *p* = 0.019), whereas ΔRBI showed an inverse association (β = – 0.281, *p* = 0.034). In addition, ΔGRI was negatively associated with changes in arm-curl performance (β = –0.316, *p* = 0.007), indicating that less favorable shifts in glutathione redox status were statistically associated with less favorable changes in upper-limb strength measures.

**Table 3:** Associations between changes in redox indices and changes in strength and functional performance outcomes in older physically active adults, stratified by sex

Outcome (Δ abs)	Predictor (Δ index)	Total Sample (*n* = 30)			Men (*n* = 20)			Women (*n* = 10)		
		β (95% CI)	p	Adj. R^2^	β (95% CI)	p	Adj. R^2^	β (95% CI)	p	Adj. R^2^
Handgrip (kg)	Δ ACI	− 0.08 [− 0.334, 0.175]	0.533	0.115	− 0.095 [− 0.42, 0.231]	0.559	0.101	− 0.074 [− 0.583, 0.434]	0.761	0.004
	Δ ODI	− 0.03 [− 0.277, 0.217]	0.809	0.122	− 0.098 [− 0.41, 0.214]	0.529	0.102	0.032 [− 0.507, 0.572]	0.900	0.022
	Δ GRI	− 0.165 [− 0.426, 0.095]	0.209	0.134	− 0.113 [− 0.448, 0.221]	0.496	0.104	− 0.337 [− 0.826, 0.152]	0.163	0.116
	Δ RBI	− 0.046 [− 0.303, 0.21]	0.718	0.123	0.014 [− 0.314, 0.342]	0.932	0.092	− 0.07 [− 0.646, 0.507]	0.800	0.025
Leg/Back (kg)	Δ ACI	− 0.194 [− 0.437, 0.049]	0.115	0.192	− 0.341 [− 0.633, − 0.048]	**0.024**	0.271	− 0.029 [− 0.517, 0.459]	0.902	0.083
	Δ ODI	0.085 [− 0.156, 0.325]	0.483	0.173	0.062 [− 0.24, 0.364]	0.678	0.162	0.211 [− 0.279, 0.702]	0.373	0.192
	Δ GRI	− 0.145 [− 0.4, 0.11]	0.259	0.175	− 0.195 [− 0.513, 0.122]	0.221	0.193	− 0.105 [− 0.6, 0.389]	0.658	0.094
	Δ RBI	− 0.241 [− 0.483, 0.001]	0.051	0.222	− 0.32 [− 0.617, − 0.023]	0.035	0.257	− 0.18 [− 0.71, 0.35]	0.480	0.175
Arm curl (kg)	Δ ACI	− 0.086 [− 0.319, 0.148]	0.466	0.252	− 0.166 [− 0.446, 0.113]	0.235	0.336	− 0.055 [− 0.566, 0.456]	0.823	− 0.006
	Δ ODI	0.078 [− 0.149, 0.304]	0.495	0.265	0.063 [− 0.21, 0.336]	0.642	0.313	0.091 [− 0.447, 0.63]	0.723	0.024
	Δ GRI	− 0.316 [− 0.545, − 0.088]	**0.007**	0.337	− 0.474 [− 0.72, − 0.227]	** < 0.001**	0.514	0.049 [− 0.473, 0.57]	0.845	− 0.007
	Δ RBI	− 0.142 [− 0.374, 0.091]	0.228	0.278	− 0.185 [− 0.465, 0.094]	0.187	0.342	− 0.12 [− 0.696, 0.455]	0.662	0.029
Push − ups 30 s (reps)	Δ ACI	− 0.052 [− 0.262, 0.157]	0.619	0.397	− 0.139 [− 0.392, 0.114]	0.273	0.455	− 0.15 [− 0.511, 0.212]	0.393	0.497
	Δ ODI	− 0.073 [− 0.273, 0.126]	0.463	0.429	− 0.093 [− 0.338, 0.153]	0.449	0.445	0.111 [− 0.262, 0.484]	0.535	0.532
	Δ GRI	− 0.093 [− 0.31, 0.123]	0.391	0.403	− 0.154 [− 0.414, 0.107]	0.240	0.458	− 0.195 [− 0.558, 0.168]	0.272	0.512
	Δ RBI	0.015 [− 0.193, 0.223]	0.886	0.424	− 0.025 [− 0.284, 0.233]	0.844	0.437	− 0.256 [− 0.635, 0.123]	0.170	0.578
Crunches 30 s (reps)	Δ ACI	0.026 [− 0.209, 0.261]	0.828	0.244	− 0.15 [− 0.446, 0.146]	0.310	0.254	0.23 [− 0.222, 0.682]	0.296	0.213
	Δ ODI	0.019 [− 0.21, 0.248]	0.871	0.247	− 0.122 [− 0.408, 0.164]	0.392	0.248	0.328 [− 0.134, 0.791]	0.151	0.281
	Δ GRI	− 0.042 [− 0.286, 0.201]	0.728	0.245	− 0.217 [− 0.519, 0.084]	0.152	0.275	0.301 [− 0.149, 0.751]	0.175	0.25
	Δ RBI	0.005 [− 0.233, 0.243]	0.965	0.247	− 0.008 [− 0.31, 0.294]	0.959	0.232	0.023 [− 0.509, 0.554]	0.929	0.171
Isometric bar (s)	Δ ACI	0.063 [− 0.17, 0.296]	0.591	0.258	0.028 [− 0.258, 0.315]	0.842	0.299	0.064 [− 0.429, 0.558]	0.785	0.06
	Δ ODI	− 0.194 [− 0.414, 0.027]	0.084	0.303	− 0.175 [− 0.445, 0.095]	0.196	0.331	− 0.311 [− 0.804, 0.181]	0.198	0.185
	Δ GRI	− 0.117 [− 0.357, 0.123]	0.332	0.267	− 0.182 [− 0.472, 0.107]	0.210	0.329	− 0.039 [− 0.544, 0.465]	0.870	0.057
	Δ RBI	0.218 [− 0.01, 0.445]	0.061	0.31	0.178 [− 0.104, 0.461]	0.208	0.329	0.386 [− 0.13, 0.902]	0.132	0.219
SPPB score	Δ ACI	− 0.029 [− 0.296, 0.238]	0.828	0.023	0.21 [− 0.121, 0.54]	0.206	0.071	− 0.219 [− 0.709, 0.271]	0.358	0.075
	Δ ODI	0.299 [0.052, 0.547]	**0.019**	0.121	0.34 [0.036, 0.644]	0.029	0.151	0.054 [− 0.451, 0.559]	0.823	0.143
	Δ GRI	0.023 [− 0.254, 0.3]	0.871	0.023	0.151 [− 0.194, 0.496]	0.381	0.05	− 0.171 [− 0.677, 0.334]	0.483	0.053
	Δ RBI	− 0.281 [− 0.54, − 0.022]	**0.034**	0.104	− 0.141 [− 0.477, 0.195]	0.401	0.048	− 0.276 [− 0.795, 0.244]	0.276	0.208
Balance (s)	Δ ACI	0.043 [− 0.231, 0.318]	0.754	− 0.033	0.196 [− 0.153, 0.546]	0.262	− 0.039	− 0.273 [− 0.748, 0.203]	0.242	0.127
	Δ ODI	0.253 [− 0.007, 0.513]	0.056	0.031	0.438 [0.13, 0.747]	0.007	0.126	− 0.313 [− 0.82, 0.193]	0.207	0.138
	Δ GRI	− 0.022 [− 0.307, 0.263]	0.877	− 0.034	− 0.029 [− 0.396, 0.338]	0.874	− 0.075	0.096 [− 0.409, 0.601]	0.692	0.057
	Δ RBI	− 0.178 [− 0.453, 0.096]	0.198	− 0.005	− 0.238 [− 0.587, 0.11]	0.174	− 0.021	− 0.073 [− 0.644, 0.498]	0.789	0.043
4-m walk time (s)	Δ ACI	0.051 [− 0.199, 0.301]	0.683	0.146	0.003 [− 0.316, 0.323]	0.984	0.131	0.199 [− 0.287, 0.686]	0.397	0.089
	Δ ODI	0.023 [− 0.221, 0.267]	0.852	0.144	0.077 [− 0.23, 0.383]	0.615	0.137	− 0.134 [− 0.663, 0.394]	0.597	0.061
	Δ GRI	− 0.142 [− 0.399, 0.114]	0.272	0.162	− 0.111 [− 0.439, 0.216]	0.495	0.142	− 0.187 [− 0.685, 0.31]	0.437	0.082
	Δ RBI	0.025 [− 0.228, 0.278]	0.844	0.144	− 0.066 [− 0.386, 0.255]	0.679	0.135	0.358 [− 0.178, 0.894]	0.175	0.156
Chair-stand time (s)	Δ ACI	0.131 [− 0.095, 0.357]	0.252	0.301	0.091 [− 0.192, 0.373]	0.519	0.321	0.195 [− 0.254, 0.644]	0.371	0.224
	Δ ODI	− 0.115 [− 0.335, 0.104]	0.298	0.309	− 0.065 [− 0.337, 0.208]	0.633	0.317	− 0.079 [− 0.519, 0.362]	0.709	0.348
	Δ GRI	− 0.059 [− 0.295, 0.178]	0.622	0.287	− 0.054 [− 0.347, 0.238]	0.709	0.315	− 0.09 [− 0.558, 0.377]	0.687	0.191
	Δ RBI	0.212 [− 0.011, 0.435]	0.062	0.339	0.128 [− 0.154, 0.411]	0.363	0.329	0.261 [− 0.191, 0.712]	0.237	0.402
TUG (s)	Δ ACI	− 0.118 [− 0.363, 0.127]	0.337	0.178	0.067 [− 0.238, 0.372]	0.657	0.210	− 0.334 [− 0.821, 0.154]	0.166	0.085
	Δ ODI	− 0.186 [− 0.422, 0.05]	0.121	0.199	− 0.112 [− 0.404, 0.179]	0.441	0.219	− 0.391 [− 0.907, 0.125]	0.127	0.106
	Δ GRI	− 0.084 [− 0.34, 0.171]	0.511	0.171	0.019 [− 0.296, 0.334]	0.905	0.206	− 0.287 [− 0.793, 0.22]	0.248	0.05
	Δ RBI	0.057 [− 0.194, 0.307]	0.652	0.166	0.152 [− 0.151, 0.455]	0.315	0.228	− 0.078 [− 0.674, 0.518]	0.784	− 0.044
400 m walk (min)	Δ ACI	− 0.097 [− 0.323, 0.129]	0.394	0.300	0.136 [− 0.132, 0.403]	0.311	0.391	− 0.393 [− 0.841, 0.054]	0.081	0.228
	Δ ODI	0.085 [− 0.137, 0.307]	0.445	0.292	0.226 [− 0.024, 0.475]	0.075	0.426	− 0.338 [− 0.848, 0.173]	0.179	0.125
	Δ GRI	− 0.049 [− 0.285, 0.186]	0.676	0.293	0.104 [− 0.174, 0.382]	0.451	0.383	− 0.353 [− 0.82, 0.114]	0.129	0.191
	Δ RBI	− 0.157 [− 0.384, 0.071]	0.174	0.309	− 0.096 [− 0.367, 0.175]	0.477	0.382	− 0.195 [− 0.766, 0.376]	0.478	0.043

Values are standardized regression coefficients (β) with 95% confidence intervals from linear models adjusted for age and supplementation group. Adj. R^2^: adjusted coefficient of determination. Analyses are presented for the total sample, men, and women separately. Significant associations (*p* < 0.05) are indicated in bold

Sex-stratified models suggested partially divergent patterns. In men, ΔGRI remained negatively associated with arm-curl performance (β = – 0.474, *p* < 0.001), and ΔACI was inversely related to changes in leg/back strength (β = – 0.341, *p* = 0.024). ΔRBI also showed a trend towards an inverse association with chair-stand time (β = – 0.212, *p* = 0.062). In women, no statistically significant associations emerged, although estimates were imprecise due to higher variability and the smaller sample size (*n* = 10).

These analyses were not adjusted for multiple testing and should be interpreted as hypothesis-generating. Overall, the pattern suggests that inter-individual variability in composite redox indices, particularly glutathione-based measures, may statistically co-vary with changes in selected functional measures in physically active older adults, but confirmation in larger, adequately powered samples specifically designed to test these relationships is required.

## Discussion

This randomized, double-blind, placebo-controlled crossover trial examined the effects of creatine plus β-hydroxy-β-methylbutyrate (CRE + HMB) supplementation on systemic oxidative stress biomarkers in physically active older adults, and explored how redox adaptations relate to previously published functional changes from the parent trial. Importantly, physical function outcomes were not analyzed as efficacy endpoints in this study, consistent with the methodological framework described in Sect. “Muscle strength and functional performance”. Overall, most biomarkers remained stable across conditions, whereas glutathione-related parameters and lipid peroxidation showed condition-specific patterns. Because these effects did not withstand FDR correction, they should be interpreted as exploratory.

Aging is characterized by a progressive disruption of redox homeostasis, driven by elevated ROS production and impaired antioxidant defenses (Ji 2015; Candow et al. 2021). In this context, nutritional strategies capable of stabilizing redox status may help preserve muscle function—a notion supported by experimental research demonstrating oxidative stress–induced declines in contractile performance (Fulle et al. 2004; Powers et al. 2016). In the present study, classical biomarkers such as FRAP, protein carbonyls, and GPx remained stable across conditions, whereas GSSG and MDA demonstrated condition-specific changes. This selective sensitivity is consistent with prior work identifying the glutathione system as one of the most responsive redox buffers in aging muscle (Ji 2015; Sies 2015).

lthough CRE + HMB effects on composite indices (particularly GRI) showed nominal between-group patterns, these differences did not survive FDR correction, suggesting that the intervention exerted, at most, a moderate modulatory influence on the glutathione axis. This interpretation aligns with mechanistic evidence demonstrating creatine’s direct antioxidant actions (Sestili et al. 2006, 2011) and HMB’s cytoprotective and anti-inflammatory effects (Fitschen et al. 2013; Fernández-Landa et al. 2024).

MDA was quantified using the TBARS assay, a widely used but non-specific method susceptible to interference from multiple aldehydes and other chromogens, which may lead to overestimation of lipid peroxidation compared with more specific markers such as F₂-isoprostanes (Pisoschi et al. 2021). Although PRE–POST paired samples were analyzed on the same plate to reduce variability, the lack of absolute specificity of TBARS must be acknowledged. Similarly, the GSH/GSSG ratio is highly sensitive to sampling time, delayed processing, and freeze–thaw cycles (Jones 2006); although our single-freeze–thaw protocol mitigates these risks, analytical constraints remain a key factor when interpreting the dissociation between stable classical markers and responsive glutathione metrics. Taken together, the attenuation of the GSSG rise under supplementation is compatible with creatine’s documented antioxidant and membrane-stabilizing actions, whereas the isolated increase in MDA observed in men may reflect HMB-related, sex-dependent modulation of inflammatory and lipid peroxidation pathways.

Inter-group comparisons suggested a potential attenuation of training-related perturbations in glutathione homeostasis with CRE + HMB. Under placebo, GSSG increased from PRE to POST, whereas supplementation appeared to attenuate this rise. This divergence was most evident in men, indicating that GSSG and GRI may be sensitive indicators of training-induced redox stress and its nutritional modulation. Conversely, MDA increased in men under supplementation. The most straightforward interpretation of this finding is a potential pro-oxidant response to CRE + HMB in this subgroup. Because the interaction did not survive FDR correction and the study was not powered for sex-specific analyses, this result should be considered an unexpected and preliminary observation. Although a hormetic redox response cannot be fully excluded—particularly in the context of training-induced metabolic demand—this explanation remains speculative and cannot be supported by the present data. Importantly, the TBARS assay is non-specific and susceptible to analytical interference, limiting the ability to infer true lipid peroxidation (Grotto et al. 2009). Future trials incorporating more specific lipid oxidation markers—such as F₂-isoprostanes, considered the gold standard for in vivo lipid peroxidation (Milne et al. 2005)—and adequately powered sex-stratified analyses are required to clarify the biological meaning of this pattern.

The integration of biomarkers into composite indices (ACI, ODI, GRI, RBI) represents a novel dimension of this study. While single biomarkers provide narrow snapshots of redox biology, composite indices may capture systemic redox behavior more effectively (Jones 2006; Pisoschi et al. 2021). Regressions revealed that ΔGRI was inversely associated with changes in arm-curl performance, consistent with mechanistic links between glutathione equilibrium and muscle contractility (Powers et al. 2011; Ji 2015). Similarly, ΔODI and ΔRBI correlated with SPPB, a validated indicator of functional capacity (Cruz-Jentoft et al. 2019). Because functional data in this study were used exclusively as exploratory correlates (not outcomes), these associations should not be interpreted as evidence of supplementation efficacy.

Comparisons with previous supplementation trials help contextualize our findings. Traditional antioxidant vitamins have yielded inconsistent effects in older adults (Ji 2015; Pisoschi et al. 2021), possibly due to limited bioavailability or interference with adaptive redox signaling. In contrast, CRE + HMB combines ergogenic, cytoprotective, and anti-catabolic mechanisms: creatine enhances phosphocreatine turnover and may reduce ROS formation (Sestili et al. 2006, 2011; Candow et al. 2019), while HMB reduces proteolysis and modulates NF-κB–linked inflammatory pathways (Aversa et al. 2011; Fitschen et al. 2013). Studies in athletes have shown improved performance, reduced muscle damage, and favorable hormonal modulation with this combination (Fernández-Landa et al. 2019, 2020a, b). Our findings add preliminary evidence of redox-related effects in older adults, though confirmatory trials are required.

Sex-dependent differences—such as higher MDA in men and greater redox variability in women—mirror documented sex differences in mitochondrial function, inflammatory tone, and oxidative stress biology (Brunelli et al. 2014; Ostan et al. 2016; Kander et al. 2017). While underpowered for formal interactions, these tendencies reinforce the importance of sex-specific analyses in future work.

The strengths of this study include its randomized double-blind crossover design, excellent adherence, stringent PRE–POST sample handling, and a comprehensive biomarker panel incorporating composite indices. However, limitations must be acknowledged. First, sample size—particularly among women—restricted power for detecting subtle or sex-specific effects. Second, the six-week intervention may not capture longer-term redox or functional adaptations. Third, plasma biomarkers reflect systemic, not muscle-specific, redox status. Fourth, although carryover testing was performed, small crossover trials may lack sensitivity to detect subtle carryover effects (Senn 2003; Wellek and Blettner 2012; Jones and Kenward 2014). Fifth, the TBARS assay limits interpretation of the lipid peroxidation results. Finally, while FDR correction reduced false positives, the small sample size may have increased the risk of false negatives.

Despite these limitations, the consistency of nominal improvements in GSSG-based metrics, along with exploratory associations between composite redox indices and functional changes, suggests that CRE + HMB may modulate glutathione-centered redox pathways in physically active older adults. In conjunction with previous work using the same supplementation protocol (Ramos-Hernández et al. 2025), these findings support further investigation into CRE + HMB as a potential adjunct to exercise for promoting redox stability and functional resilience during aging.

## Conclusions

In physically active older adults, six weeks of creatine plus β-hydroxy-β-methylbutyrate (CRE + HMB) supplementation were associated with nominal changes in glutathione-based redox markers, particularly by preventing the exercise-associated rise in GSSG and preserving the Glutathione Redox Index (GRI). Although most effects did not remain significant after correction for multiple comparisons, the overall pattern is consistent with a potential role of CRE + HMB in modulating systemic redox balance during training. Exploratory analyses indicated that changes in composite redox indices co-varied with selected functional measures, with possible sex-specific patterns; however, these findings must be interpreted with caution given the limited sample size and absence of multiplicity adjustment.

Taken together, these results provide preliminary human evidence that combined creatine and HMB supplementation may modulate redox homeostasis in active older adults. Larger and longer-duration trials—including sex-balanced samples and more specific oxidative stress endpoints—are needed to confirm these findings and clarify their physiological relevance. In conjunction with previous work using the same supplementation protocol (Ramos-Hernández et al. 2025), the present results support continued investigation of CRE + HMB as a potential adjunct to exercise for supporting healthy aging.

## Data Availability

No datasets were generated or analysed during the current study.
